# Advances and considerations in AD tau-targeted immunotherapy

**DOI:** 10.1016/j.nbd.2019.104707

**Published:** 2019-12-10

**Authors:** Alice Bittar, Nemil Bhatt, Rakez Kayed

**Affiliations:** a Department of Neurology, The Mitchell Center for Neurodegenerative Diseases, University of Texas Medical Branch, 301 University Blvd, Galveston, TX 77555, United States of America; b Department of Neuroscience, Cell Biology and Anatomy, Graduate School of Biomedical Sciences, University of Texas Medical Branch, 301 University Blvd, Galveston, TX 77555, United States of America

**Keywords:** Tau-targeted immunotherapy, Tau, Amyloid Beta immunotherapy, Alzheimer’s disease, Biomarkers, extracellular tau, Alzheimer’s disease clinical trials

## Abstract

The multifactorial and complex nature of Alzheimer’s disease (AD) has made it difficult to identify therapeutic targets that are causally involved in the disease process. However, accumulating evidence from experimental and clinical studies that investigate the early disease process point towards the required role of tau in AD etiology. Importantly, a large number of studies investigate and characterize the plethora of pathological forms of tau protein involved in disease onset and propagation. Immunotherapy is one of the most clinical approaches anticipated to make a difference in the field of AD therapeutics. Tau –targeted immunotherapy is the new direction after the failure of amyloid beta (Aß)-targeted immunotherapy and the growing number of studies that highlight the Aß-independent disease process. It is now well established that immunotherapy alone will most likely be insufficient as a monotherapy. Therefore, this review discusses updates on tau-targeted immunotherapy studies, AD-relevant tau species, updates on promising biomarkers and a prospect on combination therapies to surround the disease propagation in an efficient and timely manner.

## Graphical Abstract

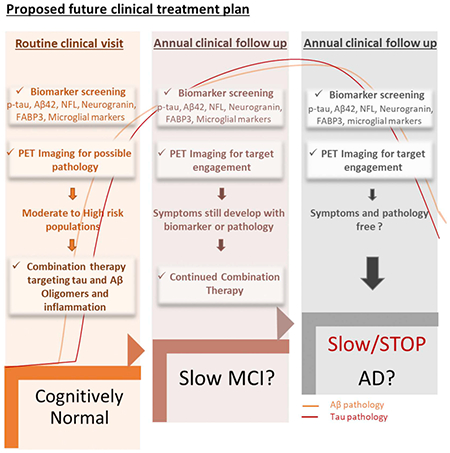

## 1. Introduction

Despite all the failed immunotherapy clinical trials in Alzheimer’s disease (AD), amyloid beta (Aß) and tau targeted immunotherapies are still at the forefront of therapeutic approaches (Hoskin et al., 2019; Cummings et al., 2019). Besides being the identifying hallmarks of the disease, Aß and tau aggregates have been extensively studied and directly linked to neurodegeneration. Aß and tau, primarily the oligomeric species of each, have been shown to be neurotoxic both extracellularly and intracellularly (Frost et al., 2009; Sebastián-Serrano et al., 2018). Aß and tau oligomers have been shown to disrupt membrane and synaptic integrity as well as calcium balance, long term potentiation, cellular cytoskeleton and, most importantly, synaptic spines and synaptic communication that leads to progressive cognitive decline (Polanco et al., 2017). The role of tau is more agreed upon compared to the controversy around the consequential role of Aß in the disease process. Nevertheless, there is evidence that suggests a synergistic toxic effect between Aß and tau aggregates which might be exploited by immunotherapy (Pascoal et al., 2017).

Some studies suggest that Aß malfunction lies upstream of tau malfunction and triggers tau pathology (Bloom, 2014; Hurtado et al., 2010; Lewis et al., 2001; Götz et al., 2001). However, targeting Aß by immunotherapy did not lead to a decrease in tau pathology nor slowed down cognitive decline in clinical trials (Panza et al., 2019; Medina, 2018). Targeting Aß was shown to be ineffective in early intervention studies in patients with mild cognitive impairment (MCI) and prodromal Alzheimer’s disease (AD) (Cummings et al., 2018). Other studies supporting this idea have shown that Aß pathology itself is not linked to the neurodegeneration and dementia observed (Giacobini and Gold, 2013) but rather, AD development and the associated neurodegeneration correlate to APP malfunction (Lewczuk et al., 2018). Moreover, APP has been shown to be involved in keeping synaptic and axonal integrity (Rusu et al., 2007) and in intracellular transport (Rodrigues et al., 2012; Kametani and Hasegawa, 2018) In addition, APP fragments have been shown to disrupt synaptic plasticity and cellular metabolism, and accumulate in dystrophic neurites (Kametani and Hasegawa, 2018) in both sporadic and familial AD. To add, APP malfunction has been closely linked to tau phosphorylation, aggregation and accumulation (Takahashi et al., 2015). Therefore, all the above support the notion that APP, and not Aß, along with tau are the main drivers of AD.

While there are continuous efforts on identifying novel drug targets along the APP metabolism pathway, APP metabolism-targeting therapeutic methods remain far from being established. (Takahashi et al., 2015). On the other hand, tau malfunction correlates with the onset and propagation of AD pathology. In addition, tau load in the brain correlates with cognitive decline, and removal of tau aggregates, in their different forms, have attenuated pathology spread and cognitive decline in animal models. For these reasons, tau-targeted immunotherapy is on the rise. This short review will cover: 1) the latest updates on the current on-going tau-targeted immunotherapy clinical trials, 2) targeting the different pathological forms of tau, 3) the journey to identify biomarkers that will aid in early disease detection and thus early immunotherapy intervention, and 4) a futuristic look onto possible combinational immunotherapy approaches.

## 2. Update on ongoing Immunotherapy clinical trials

One of the major reasons believed to be behind the failure of Aß-targeted immunotherapy clinical trials is that Aß load in the brain does not correlate with the level of dementia. Neither reducing Aß plaques nor oligomers has helped in slowing down neurodegeneration. Therefore, the general conclusion was that Aß accumulation is irrelevant to neurodegeneration and might be just a consequence of the disease process (Panza et al., 2019).

Given that tau pathology follows the Braak and Braak staging of AD and correlates well with the timeline of neurodegeneration and dementia progression (Lowe et al., 2017), outcomes with tau immunotherapy appear promising. In addition, several types of dementia are now classified as tauopathies including AD. That is because these disorders all share tau malfunction, and misfolded tau propagation is a prominent feature of the disease stages and overall pathology. Knowing that tau is a microtubule binding protein and is therefore directly involved in the cellular integrity, i.e. is not a byproduct of a mutation like Aß, tau malfunction is more likely to be causally linked to neurodegeneration. To add, misfolded forms of tau have been shown to be toxic, able to seed normal forms of tau and propagate the pathology. Therefore, stopping the propagation of abnormal tau is likely to have more beneficial functional effects than targeting non-toxic aggregates of cellular byproducts like Aß.

Given the above, tau immunotherapy is now in clinical trials. At this stage, several tau species related to neurodegeneration and dementia have been discovered and characterized. Also, several humanized tau antibodies have also been produced and are currently being tested in clinical trials. AADvac-1 (NCT02031198) is the first in-man active tau antibody to go into phase I clinical trials in 2016 (Novak et al., 2017; Novak et al., 2018a, 2018b). It had very favorable safety profiles and resulted in less cognitive decline and brain atrophy in patients with mild to moderate AD. AADvac-1 is currently in phase II trials awaiting disease modifying results in patients with mild AD. In September 2019, results of phase II were announced in a press release by Axon Neuroscience. AADvac-1 showed a promising safety profile and immunogenicity in phase II with no significant adverse events observed between immunized and placebo patients. Interestingly, AADvac-1 also showed a statistically significant decrease in blood (NfL) and CSF (pTau181 and pTau217) biomarkers, bringing patients’ biomarkers levels close to normal. This strongly suggests that AADvac-1 is targeting and affecting tau pathology. The press release also mentioned a cognitive improvement among the young population only.

ABBV-8E12 is another tau antibody in phase II clinical trials (NCT02880956). 8E12 is a monoclonal tau antibody that targets extracellular pathological tau aggregates. Results were very promising as 8E10 was shown to target larger insoluble tau aggregates and reduce tau seeding and propagation. 8E12 has also showed favorable safety profiles in progressive supranuclear palsy (PSP) patients and it has entered phase II clinical trial which will evaluate the long-term safety and tolerability in PSP and mild AD patients, as well as evaluate the ability of the antibody to delay cognitive decline. Phase II trial on PSP patients was terminated in July of 2019 after failed a futility assessment. Experts in the field report that this result was expected due to the old age of the patients (Alzforum, 26 Jul 2019). However, phase II on MCI and early AD patients is still ongoing without changes. The study is projected to finish in 2021 (West et al., 2017).

Another monoclonal antibody, however targeting a different form of tau, is BIIB092 (NCT02460094) (also IPN007 or BMS-986168). This humanized antibody targets extracellular N-termini of fragmented tau. This antibody also showed favorable safety profiles in phase I trial in PSP patients and reductions in cerebral spinal fluid (CSF) free fragmented tau levels (Qureshi et al., 2018; Dam et al., 2018). It is now enrolled in a phase II trial in MCI and mild AD patients (NCT03068468). The clinical trial is projected to finish in 2020 (Ratti et al., 2018; Boxer et al., 2019).

Last but not least, RO7105705 is a monoclonal tau-antibody in phase II trial (NCT02820896). Since it is reported to target the N-terminus of all six extracellular human tau isoforms, in both monomeric and oligomeric forms, it appears to be very promising. After showing a favorable safety profile, RO7105705 antibody is in two clinical trials on “probable AD” patients, i.e., based on PET imaging or CSF Aß42 levels. The two trials aim at measuring tau in the brain and cognitive performance at three different doses in comparison to placebo. The studies are projected to finish by 2020 and 2021 (NCT03289143) (Kerchner et al., 2017).

Several other tau antibodies have also entered the clinical trials marathon. One interesting antibody currently in phase I is reported to bind and remodel both Aß and tau misfolded aggregates. This antibody, NPT088, was shown to reduce Aß and p-tau pathology (Levenson et al., 2016). It was also shown to notably reverse brain atrophy and cognitive deficits in aged Tg2576 hAPP mice (Levenson et al., 2016). Overall, the promising aspect about this race of clinical trials is that almost each of the utilized antibodies targets a different tau epitope. However, concurrently, most of the antibodies that made it to clinical trials, target the N-terminus of a form of extracellular tau. With the disease etiology being more supportive of tau involvement, and with more studies aiming at identifying AD-relevant pathological tau species, tau immunotherapy is more promising than any time before (Table 1).

## 3. AD-relevant tau species between hyperphosphorylated and conformational aggregates

### 3.1. Hyperphosphorylated tau

In AD, tau pathology is described as insoluble intracellular neurofibrillary tangles (NFTs) consisting of hyper-phosphorylated and aggregated tau species. Studies have shown, however, that the smaller soluble tau aggregates are the toxic tau species underlying the spread of pathology and neurodegeneration (Guerrero-Muñoz et al., 2015; Shafiei et al., 2017; Fá et al., 2016). Those species mainly consist of hyperphosphorylated and misfolded tau conformations (oligomers, seed-competent monomers) (Mirbaha et al., 2018; Wu et al., 2017). As mentioned earlier, the population of pathological tau species in AD is very heterogeneous. This heterogeneity is currently an important obstacle in the face of tau-targeted immunotherapy. The success of immunotherapy in AD and other tauopathies greatly depends on the clear characterization of most of the pathological tau species. There are three main differences between pathological and physiological tau. First, the ability to acquired toxicity due to loss of function/gain of toxic function. Second, seed competency and the distinct epitopes that get exposed due to misfolding and third, lead to aberrant aggregation. It was recently shown that the exposure of an aggregation-prone domain due to misfolding renders tau conformations seed competent (Dujardin et al., 2018; Mirbaha et al., 2018) However, what renders a tau conformation toxic is still undetermined, aside from the loss of normal function. Hyperphosphorylation, on one hand, is one form of toxic loss of function due to its direct role in tau detachment from the microtubules. Hyperphosphorylation is shown to precede or initiate tau misfolding in AD human brains (Tai et al., 2014). In turn, misfolded soluble tau spreads from cell-to-cell in a neuronal network dependent pattern starting in the entorhinal cortex, going through the hippocampus and reaching the isocortex (Kaufman et al., 2018; Neddens et al., 2018). This transcellular tau spreading and seeding competency highlights the importance of immunotherapy as a leading therapeutic approach in removing/ neutralizing misfolded tau species as well as stopping the spreading and seeding processes.

Aside from the tau-targeted antibodies that are currently in clinical trials, many studies are currently characterizing novel AD-relevant tau species and epitopes as immunotherapy targets. Reviewing most of the recent studies on tau-relevant species and immunotherapies, the complex heterogeneity of AD-relevant pathologic tau species becomes clearer and clearer. However, a few AD-relevant tau species stand out as the most promising immunotherapy targets. This section will highlight the most recent and therapeutically promising pathological species in animal models.

Some phospho-epitopes have been specifically and directly linked to AD. An interesting study by Neddens et al., (2018) characterized a group of the most commonly studied phospho-epitopes in terms of their levels over the course of the disease, i.e. over the Braak stages. All the investigated sites increased gradually over the course of the disease, however, pSer396 and pSer422 were specifically hyperphosphorylated at earlier disease stages. Characterizing early disease stage-relevant tau species is a key step-forward in targeting toxic species early on, before full disease pathology and cognitive decline.

Recently, the first crystal structure of C5.2, a monoclonal antibody that is specific to pSer396 epitope was generated. This antibody recognizes the middle region of tau where the microtubule binding domain (MTBD) is located (Perdersen et al., 2017; Chukwu et al., 2018). Further analysis revealed that the pSer396 phosphorylation site leads to a switch from alpha-helix to β-strand motif that misfolds tau and stabilizes a toxic conformation (Chukwu et al., 2018). Early prevention of pSer396 phosphorylation by immunotherapy is potentially promising to prevent propagation of this pathological tau species. C5.2 is the only antibody that targets the pSer396 site (C terminal epitope). PHF1, a monoclonal antibody that recognizes pSer396/Ser404 epitope, has been heavily tested using passive immunotherapy. PHF1 was tested in P301S, P301L, htau/PS1 and rTg4510 mouse models (Liu et al., 2016; Boutajangout et al., 2010; Chai et al., 2011; d’Abramo et al., 2013; Shahpasand et al., 2018). However, results showed that PHF1 immunization is mostly effective in reducing motor rather than cognitive decline. This highlights the difficulty in reversing cognitive and memory deficits after a certain point in the course of the disease. In other words, these results also reassure that early intervention with immunotherapy, before the onset of symptoms, is necessary to see promising effects on mouse cognitive behavior. These studies raise the question of whether C5.2 antibody will be effective in reducing cognitive decline when tested in immunotherapy studies. Also, keeping in mind that different tau epitopes get hyperphosphorylated along the disease spectrum (Neddens et al., 2018). Therefore, other questions arise as to whether early immunotherapy interventions against early phosphorylation sites helps in reducing the formation of other phosphorylated species that appear later over the course of the disease (Chukwu et al., 2018).

Another antibody, TWF9, recognizes beta-sheet conformations on tau oligomeric species (Herline et al., 2018). This antibody was shown to be specific to tau species in human AD and MCI samples. Detailed epitope description was not provided in this study; however, it was also shown to be specific to Aß oligomeric species as well as PHFs. Intriguingly, a short-term administration of TWF9 lead to reduced memory deficits as well as reduced soluble levels of phosphorylated tau on 3xTg-AD old mice (18–22 months). To date, this is one of the few studies showing disease-course modifying or late intervention passive immunotherapy against soluble p-tau species.

pT231 is another phospho-tau epitope that is highly implicated in earlier stages of AD-relevant neurodegeneration (Albayram et al., 2016; Nakamura et al., 2013). pT231 phospho-tau monomer has been shown to be seed competent. It also colocalizes with pathogenic oligomeric species and hyperphosphorylated PHF species as well as AT8 and Alz50-recognized species (Luna-Munoz et al., 2007; Shahpasand et al., 2018; Kanaan et al., 2016). These findings suggest that immunotherapy targeting pT231 tau might be effective. PHF-6, a monoclonal antibody specific to pT231 was evaluated in rTg4510 mouse model alongside PHF13 (pSer396) (Sankaranarayanan et al., 2015). Both antibodies showed reduction in soluble but not insoluble forms of tau. This reduction was accompanied with NOR memory cognitive improvement. However, again, the antibodies were administered in early disease stages.

pT231 exists in two isomeric conformations, cis and trans, due to the presence of a proline residue near the phosphorylation site. Cis pT231 tau (cis p-tau) is implicated more in neurodegeneration than trans p-tau. It is even suggested that cis p-tau is a very early disease marker, perhaps the first phosphorylated form of pathological tau, however, only in TBI-induced neurodegeneration (Kondo et al., 2015). cis mAb, a monoclonal cis p-tau antibody, have been shown to block neuronal toxicity and improve cortical based behavioral functions like decision making and risk taking (Kondo et al., 2015). Nevertheless, cis p-tau have not been implicated in AD-specific studies (Albayram et al., 2018; Naserkhaki et al., 2019; Albayram et al., 2017). Overall, these results highlight the promising therapeutic potential of targeting pT231 epitope. Further characterization, however, is needed to definitively determine its role (particularly cis p-tau) in AD-relevant neurodegeneration.

In addition, according to the study done by Neddens et al. (2018), pT231 is high in level at early disease stages in the entorhinal cortex only. Nevertheless, pT231 levels do not increase in the isocortex except until Braak 4 stage (Neddens et al., 2018), which suggests that pT231 might not be as reflective of AD disease progression as other early phosphorylation sites like pSer396 for example.

### 3.2. Toxic conformations

Toxic conformations of tau, on the other hand, have been heavily implicated in early disease pathology. Monoclonal antibodies like MC-1, Alz50, TOMA1 and TOMA2 have been implicated for their promising potential for immunotherapy against soluble toxic conformations and tau oligomeric species; the most toxic tau species in neurodegenerative disease including AD. Despite the non-promising pre-clinical results seen by MC-1 antibody, Eli Lilly humanized MC-1 antibody which have been tested in three clinical trials. The first two studies were phase I studies in healthy, AD, and MCI patients. A third phase II study is now active in patients with early symptomatic AD. No specific details or results have been posted about any of the studies (Jicha et al., 1997; Alam et al., 2017; Vitale et al., 2018; Schroeder et al., 2016). This continuation of clinical trials is driven most likely by the assumption/hope that early intervention will be more effective than late intervention. Another tau specific conformational antibody is the Alz50. This antibody is like MC-1 in that it recognizes a discontinuous epitope, part of the N terminal and part of the middle tau region (Jicha et al., 1997). ALz50 is shown to bind to early disease stage high molecular weight soluble tau species and not to fibrillary tau or thioflavin-positive tau.

However, N terminal targeted antibodies have been shown to be poor antibodies for AD-relevant pathological tau species. For instance, almost all the AD brain tau species are missing the N-terminal where it is shown that the N terminal is an aggregation-limiting domain. Also, N-terminal antibodies have been thought to target physiological tau in addition to truncated tau and have been shown to be not very effective in depleting the tau species pool of pathological tau species (Chukwu et al., 2018).

**Fig. 1.**
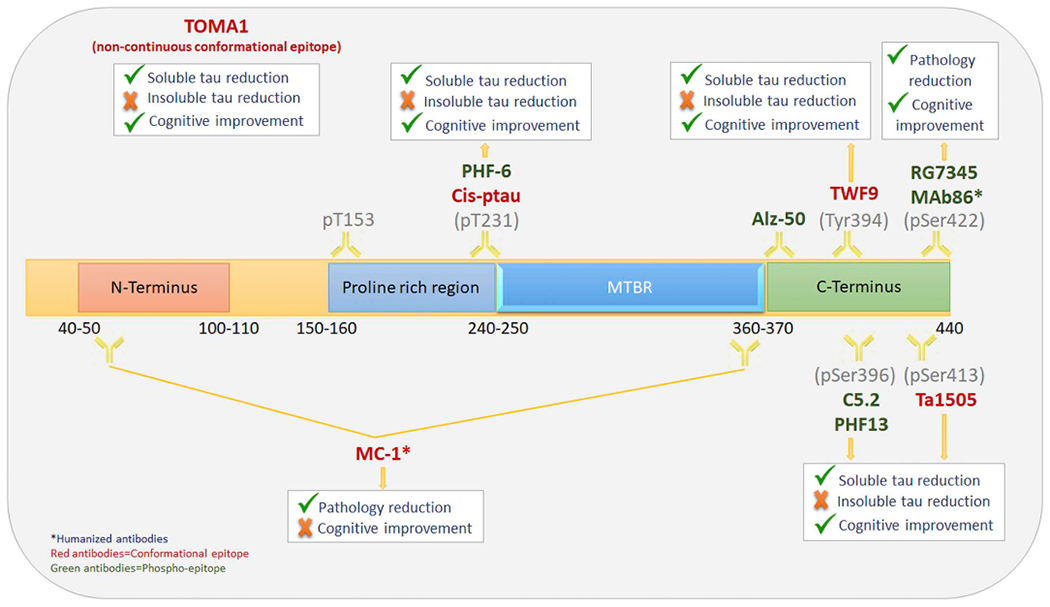
Full-length tau showing the discussed tau antibodies, their respective epitopes and their effect on soluble/insoluble tau levels and cognitive function via immunotherapy.

The MTBR, on the contrary, have been shown to be an aggregation promoting domain. Extension of tau residues in MTBR 2 and 3, due to mutations or phosphorylation, promotes beta sheet conformation and the stacking of tau misfolded monomers to form oligomers and bigger protofibrils (Zabik et al., 2017; Eschmann et al., 2015). In addition, it was shown that the C and N termini, on the other hand, are exposed in a similar fashion in both aggregated and normal tau. (Huang et al., 2018). This strongly suggests that the N and C termini are not part of the misfolding process and that antibodies targeting the MTBR or tau mid-regions might be more effective than those targeting the N or C termini in identifying pathological tau. Few antibodies that target misfolded oligomeric species are tested in immunotherapy studies. TOMA1 is one of the few antibodies that showed tau oligomers depletion from the brain and an increase of oligomers in the CSF accompanied with cognitive improvement (Castillo-Carranza et al., 2015; Kolarova et al., 2016; Sengupta et al., 2017) (Fig. 1).

All the studies mentioned above utilized passive immunotherapy approaches. Passive immunotherapy via monoclonal antibodies is the most common immunotherapy in development today due to its higher safety profiles and several other advantages over active immunotherapy (Jadhav et al., 2019). However, some studies, that proved it safe, chose to test active immunization due to its higher specificity to the pathological species targeted in the studied animal models. Active immunization in animal models have shown to be effective in reducing soluble and insoluble tau aggregates’ levels, as well as those of hyperphosphorylated tau that are believed to drive neurodegeneration and NFTs load (Sigurdsson, 2016; Sigurdsson, 2018; Theunis et al., 2013; Theunis et al., 2017). However, there are only a few studies that showed behavioral cognitive benefit along the tau reduction (Asuni et al., 2007; Asuni et al., 2007; Troquier et al., 2012).This raises many questions about whether active immunotherapy- generated antibodies are targeting the neurodegeneration-relevant tau species. Besides characterizing the neurodegeneration-relevant tau species in an animal model, active immunotherapy requires additional characterization of the relevant antibodies produced before a conclusion can be drawn out of a study. Not only that, active immunotherapy is dependent on the patients’ immune system to function in an efficient way (Siegrist and Aspinall, 2009). This produces a lot of variability due to the variable levels of functionality and immunogenicity of the patients’ immune systems in a clinical trial, and therefore produce unreliable results (Novak et al., 2018a) (Fig. 1).

### 3.3. Targeting extracellular and intracellular tau

Tau is a microtubule binding protein; therefore, it primarily exists inside the neurons under physiological conditions. Under pathological conditions, phosphorylation of tau leads to its detachment from the microtubules and its consequent loss of normal function, gain of toxic function, aggregation, and release to the extracellular space (Yamada, 2017; Kanmert et al., 2015; Magnoni et al., 2012). Tau is also released from neurons as a result of neuronal communication and neuronal death. This consequently contributes to tau aggregation and the spread of pathological tau forms (Sebastián-Serrano et al., 2018; Medina and Avila, 2014). Extracellular tau is known to exert cellular toxicity via mechanisms separate from those of intracellular tau by interacting with muscarinic cellular receptors or disrupting the cellular membrane (Gómez-Ramos et al., 2006; Yamada and Hamaguchi, 2018). However, extracellular tau is most likely rapidly degraded.

Besides the heterogeneity of pathological and non-pathological tau species (Evans et al., 2018; Frost et al., 2009; Wu et al., 2013), most antibodies are too bulky to enter the neuron for intracellular-tau clearance (Nisbet et al., 2017; Wu et al., 2018). Therefore, immunotherapy is thought to merely function through extracellular tau clearance via neutralizing available toxic tau species, blocking further transcellular spread and aggregation (McEwan et al., 2017), and clearing the antibody-tau complex either to be degraded by microglia (Funk et al., 2015) or to be released to the periphery (blood) (Jadhav et al., 2019).

Passive immunotherapy studies in animal models have shown that targeting extracellular-tau reduces intracellular tau aggregate pools. (Yanamandra et al., 2013; Gu et al., 2013; Castillo-Carranza et al., 2015). One of the explanations of this observation is the shifting of the intra-extra-cellular tau ratio balance, i.e., promoting tau release from the neurons after extracellular clearance and thus becoming available for clearance with antibodies (Castillo-Carranza et al., 2015; Umeda et al., 2015). This is highlighted in Castillo-Carranza et al., 2015, where they observe a prominent reduction in both intracellular and extracellular tau oligomeric species after tau oligomers-targeted passive immunotherapy. This reduction was accompanied by a reversal of cognitive and motor function loss in 8 month old JNPL3 mouse model. Antibody-tau oligomer complex was shown to be cleared to the blood. It was not clear, however, whether the antibodies were able to internalize into the cells, or the observed results were due to only-extracellular tau oligomers clearance. Another study by Umeda et al. applied immunotherapy in aged mice of an aggressive tauopathy model. The antibodies used targeted phospho-epitopes and reduction in intracellular tau was observed without clear evidence of antibody internalization into the neurons. That reduction in phosphorylated pathogenic tau also resulted in marked improvement in synaptic and memory functions. It is worthy to mention that the most effective antibody in this study recognized tau oligomers in addition to a phospho-epitope (Umeda et al., 2015). This point is expanded on in the combinational therapy section of the review.

A recent study by Wu et al., 2018, tracked tau antibodies in the brain and the blood of live animals via two-photon microscopy after intravenous immunotherapy (Wu et al., 2018). They reported that approximately 25–50% of the antibodies crossed the blood brain barrier and approximately 80% of it resided in the brain up until 14 days after injection. These antibodies target a phospho–tau epitope and were shown to be effective in reducing tau pathology and cognitive impairment. These antibodies were shown to internalize into the neurons via clathrin mediated endocytosis which is a very promising step towards future immunotherapy clinical trials (Congdon et al., 2013; Gu et al., 2013; Krishnaswamy et al., 2014).

Nevertheless, one of the biggest challenges faced in developing immunotherapy involves antibodies crossing the BBB and reaching their respective targets, whether that be tau or Aß. Increasing the antibody efficacy can be done through exploiting natural BBB transport systems such as carrier mediated transport (Ohtsuki and Terasaki, 2007), active efflux transport (Sanchez-Covarrubias et al., 2014) and receptor mediated transport (RMT). Pharmaceutical companies as well as academic scientists have used RMT systems to deliver therapeutic agents. One such pathway involves the transferrin receptor (TfR), where TfR is expressed in the brain endothelial cells and is responsible for maintaining brain iron homeostasis by transporting iron by endocytosis. (Barar et al., 2016; Pardridge, 2002).

Antibody production has heavily advanced and using bispecific antibodies is seeming very attractive. Bispecific antibodies are two antigen specific immunoglobulin chains combined into a single construct. This technology has been widely used in cancer therapy. Bispecific antibodies can be engineered for one arm to recognize a BBB RMT receptor and another arm to the pathological target. The RMT receptor recognizing arm permits access to the brain while the other promote the therapeutic effect. In 2014, Yu et al. demonstrated that their bispecific antibody which target the TfR receptor as well as the α-secretase, crosses into the BBB and reduces amyloid α load in nonhuman primates. The amyloid α reduction directly correlated with the amount of antibody that crosses the BBB (Yu et al., 2014). In another study, a single chain Fv antibody was fused with heavy chain chimeric antibody recognizing TfR, which reduced 60% of amyloid without increased amyloid in the blood (Weber et al., 2018). These experimental data have provided a valid strategy to overcome the BBB and deliver the necessary dose of therapeutic antibody. These strategies need to be further evaluated in clinical studies particularly trials using tau targeted immunotherapy.

Meanwhile, while multiple forms of tau aggregates are being targeted in clinical trials, a marked increase in biomarker studies for early detection of AD is taking place (Cummings et al., 2018). Tau immunotherapy clinical trials are thought to result in slowing down or preventing neurodegeneration and dementia if backed up with early detection and therefore early intervention.

## 4. Biomarkers and immunotherapy: earlier detection means earlier intervention

Previously, biomarkers were only studied through autopsy. However, in 2018, screening for biomarkers using PET/MRI imaging, CSF, or blood was added as an official diagnostic tool in clinical trials by national institute of aging (Lee et al., 2019). Not all current clinical trials demand screening for biomarkers prior to enrolling patients, however, the number of clinical trials that demand such measures is on the rise. Nevertheless, the effectiveness of including biomarker screening as a diagnostic tool in clinical trials heavily depends on the level of predictability of biomarkers to the disease stages. Current biomarkers do not predict the disease stages and there are no established biomarkers that predict AD prior to presentation of symptoms. The feasibility/ease of obtaining imaging or fluid biomarkers and the economic burden of it plays a big role in their incorporation in clinical trials (Lee et al., 2019). In addition, imaging techniques are more beneficial for disease confirmation, whereas fluid biomarkers are more beneficial for early disease detection. A combination of both is most likely needed for early disease detection, monitoring disease progression and clinical trials outcomes (Mattsson et al., 2009). So far, Aß and tau, in their different forms, are the most common targets in biomarkers studies for both early disease detection and progression (Lashley et al., 2018; Schöll et al., 2019).

The change of Aß 42 levels in CSF and its use as an AD biomarker is controversial. Opposing results have been shown by several studies (Polanco et al., 2017). However, it is well accepted that Aß 42 levels decrease in CSF as the disease progresses (Buchhave et al., 2012; Fagan et al., 2007; Olsson et al., 2016). The rationale behind it is that Aß aggregates sequester the soluble Aß 42 into larger plaques in the brain, which leaves less soluble Aß units to circulate in the CSF (Bateman et al., 2012; Lashley et al., 2018). However, if that is true, the same concept should apply to tau levels in the CSF, as well, in relation to tangle formation along the disease spectrum. However, p-tau and t-tau levels in the CSF increase with the progression of the disease. This questions whether any specific species of protein, regardless of its increase or decrease, is predictive of disease propagation (Mattsson et al., 2016; Polanco et al., 2017). T-tau increases in the CSF in several conditions in addition to neurodegeneration like in post-stroke (Hesse et al., 2001) or TBI individuals (Franz et al., 2003) and therefore might not be 100% reflective of AD disease progression (Lewczuk et al., 2018). Therefore, t-tau is not a reliable biomarker on its own. (Schraen-Maschke et al., 2008; Jadhav et al., 2019). To obtain more accurate biomarkers for AD, the field has turned to combining tau and Aß measures and calculating the ratio of tau to Aß42 in the CSF as a measure of AD pathology (Chiaravalloti et al., 2018; Fagan et al., 2007). However, all these measures reflect disease stage pathology and not preclinical pre-symptomatic stages. Therefore, they are not predictive of the disease.

New molecules, such as inflammation, neuronal lipid metabolism and synaptic function-related biomarkers have been recently identified. This is considering that inflammation and synaptic dysfunction could be two of the earliest neurodegeneration-relevant events that will precede the accumulation of pathology and disease symptoms (Fyfe, 2019; Lista and Hampel, 2017). The increase of inflammation associated sTREM2 in CSF was recently found to correlate well with tau biomarkers and AD pathology. TREM2 is one of the very well-established genetic risk factors for AD (Gratuze et al., 2018) and was recently found to correlate better with tau and not Aß pathology (Suárez-Calvet et al., 2019). FABP3, another novel potential biomarker, is a fatty acid binding protein that has been implicated in neurodegeneration and belongs to the family of APOE4. In specific, FABP3 has been shown to facilitate α-synuclein oligomerization and accumulation of Aß into plaques (Sepe et al., 2018). In addition, FABP3 levels have been shown to increase in MCI and AD patients, and to be as equally indicative of AD pathology as total and phospho-tau (Höglund et al., 2017). Neurogranin is yet another novel molecule that is promising as a predictive CSF biomarker for AD. Neurogranin is a dendritic protein that reflects the dendritic function and thus neurodegeneration. Changes in the CSF levels of neurogranin were also found to be predictive to disease progression and amyloid and tau pathology (Sepe et al., 2018). In a recent longitudinal study on old non-demented patients in Germany, FABP3 and neurogranin along with the basic AD biomarkers (p-tau, t-tau, and Aß) were measured in a cohort of cognitively healthy elderly (60–80 years old) (Höglund et al., 2017; Mattsson et al., 2016). The increase in the CSF levels of both FABP3 and neurogranin was found to significantly predict the conversion from non-AD to AD disease state. More longitudinal clinical studies are warranted to better characterize the mentioned novel biomarkers and to investigate other potential biomarkers that better predict the conversion to AD.

Moreover, it has been established that tau oligomers are most likely the most toxic and earliest state of pathological tau. Soluble tau oligomers have been shown to increase in the brain long before symptoms and pathology start (Sato et al., 2018; Maeda et al., 2006; Lasagna-Reeves et al., 2012) and correlate with the start of neuronal death and progression during disease stages more than larger or insoluble tau aggregates. However, the field lacks studies or clinical trials that look for tau oligomers in the CSF or plasma as biomarkers for disease progression. That is probably due to the difficulty of detecting those molecules in CSF or blood due to low amounts or highly variable conformations. A study by Sengupta et al. (2017), is one of the few, if not the only one looking at tau oligomers levels in CSF human samples of moderate to severe AD, mild AD, and control patients (Dorey et al., 2015; Sengupta et al., 2017). They interestingly show that tau oligomers’ levels increase in the CSF with the severity of the disease. Another study by Kolarova et al. (2016) screened serum samples from AD, MCI and control patients and found that tau oligomers in the blood decrease possibly due to impaired clearance and thus accumulate in the brain (Kolarova et al., 2016). This explains the results seen by Sengupta et al. (2017). Although the work on oligomers is not recent, the nomenclature is (Cárdenas-Aguayo et al., 2014). Strong evidence points out that oligomeric species are the most correlated with cellular neurodegeneration, disease onset, progression and therefore more studies are warranted to characterize disease related tau oligomeric species and develop methods to detect them in the CSF or blood.

CSF biomarkers are more characterized even though CSF sampling is much more invasive and cost-daunting (Mattsson et al., 2009; Zetterberg, 2019). However, in the past couple of years, there has been advancements in the field of blood biomarkers. Aß 42/40 ratios in the blood are now considered a promising predictor of the stage of dementia (Nakamura et al., 2018). The blood levels of t-tau are still controversial in terms of predictability of tau pathology and neurodegeneration in the brain, however, t-tau and p-tau are considered better blood biomarkers for AD, and the conversion from MCI to AD (Mielke et al., 2017; Zetterberg, 2017). Nevertheless, Aß, t-tau and p-tau blood levels are not considered reliable predictors of disease onset until today. Finally, plasma neurofilament light is considered, so far, the most reliable blood biomarker. It correlates well with CSF biomarkers and was recently found to predict onset of neurodegeneration in familial AD (Weston et al., 2017). However, the only problem with this biomarker is that it is not disease specific, rather it is neurodegeneration specific (Khalil et al., 2018). More studies are warranted to increase the sensitivity and replicability of blood biomarker studies in a disease specific manner. Reaching a reliable and sensitive biomarker will revolutionize the field of biomarkers and make disease detection/prediction available for everybody.

Besides CSF and blood biomarkers, PET imaging is the complementary form of biomarker detection to identify disease stages and confirm target engagement after immunotherapy. Recently, a very interesting study that aimed at using a PET tracer to parallel the stages of AD in cognitively normal, MCI and AD patients. The AD stages were successfully recapitulated by tracing tau pathology in small brain regions like amygdala, entorhinal cortex and the hippocampus. In addition, the tracer F18-AV1451 successfully discriminated tau pathology levels between normal and MCI patients, as well as between MCI and AD patients (Brier et al., 2016; Leuzy et al., 2019; Passamonti et al., 2017). This novel study is a significant advancement in the field of PET biomarkers in AD.

Overall, PET imaging could be highly utilized in combination with CSF and blood biomarkers in clinical studies in smaller and specific populations like those with TREM2 variants (Gratuze et al., 2018; Carmona et al., 2018), APOE4 carriers (Safieh et al., 2019; Shi et al., 2017) , and patients with TBI history (Gerson et al., 2016). An approach that resembles personalized medicine could be applied by utilizing PET imaging biomarkers to identify and confirm disease stage progression as well as identify higher risk populations at early disease stages (Table 2).

## 5. Combination therapy is the future

A decade of AD studies has led to one major conclusion: AD is one of the most progressive and multifactorial diseases in aging populations. The multifactorial nature of the disease stands between research and therapeutic development and makes it very difficult to stop or prevent disease progression with monotherapy approaches. Years of experimental and monotherapy clinical trials led to many advancements in understanding the complexity and timeline of the disease, identifying pathological protein species, and assessing the feasibility and risks associated with disease modifying therapies. However, most importantly, monotherapy studies have revealed the need for combination therapy approaches to overcome the complexity of the disease (Bittar et al., 2018) . A combination immunotherapy in AD would involve combining passive immunotherapy against multiple pathological proteins like Aß and tau. It is well established now that Aß and tau lie at the base of the pyramid of the AD disease process. Therefore, targeting both pathological forms of the proteins is expected to have a great therapeutic potential. Several studies also show a strong synergistic effect between the two proteins by which Aß exacerbates the toxicity of tau protein (or opposite) and promote further aggregation and propagation. This synergistic effect could be exploited in immunotherapy. Aß immunotherapy helps reduce early hyperphosphorylated tau pathology (Oddo et al., 2004). This highlights the advantage of using immunotherapy to reduce multiple related pathologies. This also further suggests a hierarchical relationship between Aß and tau and thus highlights the importance of using immunotherapy in a timely manner for the highest efficiency.

Combination therapy could also imply targeting several pathological forms of the same protein as phospho-tau and tau oligomers. Passive phospho-tau immunotherapy in general proved to be effective in reducing tau pathology and blocking phospho-tau propagation of pathology (Nisbet et al., 2017; Chai et al., 2011). However, very few studies showed an effect with targeting phospho-tau on cognitive decline (Yanamandra et al., 2013). In addition, from those few studies, only a couple phospho-antibodies were tested in aged mice and showed pathology reduction and cognitive improvement (Yanamandra et al., 2015; Dai et al., 2015). The difficulty to obtain cognitive improvement with the successful clearance of insoluble phospho-tau species suggests that the targeted tau species does not play a major role in the process of neurodegeneration leading to cognitive failure. Therefore, soluble forms of pathological tau, like tau oligomers, come into the picture due to their higher toxicity and early on detection in the disease process (Dorey et al., 2015; Sengupta et al., 2017). It is also shown that tau oligomers are found both extracellularly and intracellularly where they exert neuronal toxicity and impair synaptic function. Therefore, due to the mentioned reasons, tau oligomers form an attractive extracellular target for passive immunotherapy. A study by Umeda et al. characterized three monoclonal antibodies that target phospho-tau epitopes, one of which was chosen for an immunotherapy experiment (Ta1505 targeting pSer413) (Umeda et al., 2015). Keeping in mind that the three antibodies exhibited high affinity profiles to their respective epitopes and were very specific to tau species from AD brains, Ta1505 was the only tau antibody that recognized and reduced tau oligomers in addition to pSer413 tau. Interestingly, Ta1505 was the most effective in reversing memory deficits in comparison to age matched (14 month old) controls and clearance of tau oligomers from both intracellular and extracellular spaces. Ta1505 also restored synaptic function shown by restoring the levels of synaptic proteins (Umeda et al., 2015). This provides promising evidence towards the need of targeting more than one toxic tau species at a time to get a better effect.

## 6. Concluding thoughts

As the field is eagerly waiting on results from ongoing tau-targeted immunotherapy clinical trials, these trials will shape the next era of AD therapeutic developments. Currently, most experimental studies in animal models support the requisite role of pathological tau in AD disease onset and progression, and thus the hopes are very high for tau-targeted treatments. That being said, it is also well established that AD is a complex multifactorial chronological disease. The matter of early treatment intervention is still controversial, as well as Aβ involvement in the disease process and the direction towards Aβ-independent treatments. In addition to pathological proteins, disease cascade involves inflammation and genetic factors that feed into a vicious cycle of continuous progression and, therefore are indispensable in the treatment plan. Is early combination therapy the solution?

It is still very early to decide whether early intervention would make a difference and prevent disease progression. However, while it is well documented that when symptoms arise, the brain is already affected by synaptic loss, protein aggregation and inflammation that may not be reversible; multi approach early intervention sounds like a possible and safe venue for disease prevention. In addition, since tau pathology has been shown to start at least a decade before disease symptoms, the field now has a rough estimation on the average age at which early intervention might show promise, depending on the type of dementia. However, applying early intervention on the general population without reliable biomarkers testing preceding the start of the treatment is not practical. Therefore, early detection biomarkers stand out as a must step in testing for disease implications before early intervention. Moreover, routine early biomarker testing would be much more acceptable to the general public than straight forward early immunotherapy for example. At the moment, despite the tremendous efforts, biomarker studies are also still naïve. Nevertheless, with the promising advancements in biomarker studies, we are now closer to early disease detection and thus to early intervention.

## Figures and Tables

**Table 1:** Summary of current Alzheimer’s disease tau-targeted immunotherapy clinical trials.

Antibody	Trial I.D.	Phase	Method	Epitope	Patients	Status	End point
AADvac-1	NCT02031198	I	Active	Synthetic tau peptide	Mild AD; 50–80 years old	Safe Complete	2018
	NCT02579252	II	Active	Synthetic tau peptide	PSP, Mild to moderate AD; 50–80 years	Promising Ongoing	2019
ABBV-8E12	NCT02880956	II	Passive	Extracellular, aggregated tau	Mild AD, MCI; 50–85 years	Ongoing	2021
BIIB092	NCT03352557	II	Passive	N-terminus of tau	MCI, Mild AD and PSP; 50–80 years	Ongoing	2020
RO71015705	NCT02820896	II	Passive	Abnormal tau species	Mild to moderate AD; 50–80 years	Ongoing	2020

**Table 2:** Summery of current promising Alzheimer’s disease biomarkers in CSF/blood and PET imaging.

Biomarker	Method	Status	References
Aß 42 levels	CSF	Controversial	Buchhave et al., 2012; Fagan et al., 2007; Olsson et al., 2016
p-tau	CSF	Promising	Schraen-Maschke et al., 2008; Jadhav et al., 2019
t- tau	CSF	Controversial/Unreliable	Schraen-Maschke et al., 2008; Jadhav et al., 2019
t-tau/ Aß 42 ratio	CSF	Reliable	Chiaravalloti et al., 2018; Fagan et al., 2007
TREM2	CSF/Genetic	Promising	Gratuze et al., 2018, Suárez-Calvet et al., 2019
FABP3	CSF	Promising	Sepe et al., 2018, Höglund et al., 2017
Neurogranin	CSF	Promising	Sepe et al., 2018, Höglund et al., 2017; Mattsson et al., 2016
Tau Oligomers	CSF/Blood	Promising	Dorey et al., 2015; Sengupta et al., 2017, Kolarova et al., 2016
Aß 42/40 ratio	Blood	Reliable	Nakamura et al., 2018
neurofilament light	Blood	Reliable but not specific to Alzheimer’s	Weston et al., 2017
F18-AV1451	PET Imaging	Promising	Brier et al., 2016; Leuzy et al., 2019; Passamonti et al., 2017
